# Efficacy of radiotherapy in combination with first-line immunotherapy and chemotherapy for advanced lung squamous cell carcinoma: a propensity score analysis

**DOI:** 10.3389/fimmu.2023.1138025

**Published:** 2023-05-16

**Authors:** Jian Qin, Shouhui Yi, Hanjing Zhou, Chuan Zeng, Minghua Zou, Xuan Zeng, Zhenzhou Yang, Yusheng Huang

**Affiliations:** ^1^ Department of Oncology, the Second Affiliated Hospital of Chongqing Medical University, Chongqing, China; ^2^ Department of Oncology, the First Affiliated Hospital of Chongqing Medical University, Chongqing, China

**Keywords:** NSCLC, ICIs, LUSC, immunotherapy, radiotherapy

## Abstract

**Aim:**

To compare the efficacy and safety of radiotherapy in combination with immunotherapy after achieving disease control from the first-line combination therapy of platinum-based chemotherapy and immunotherapy for advanced lung squamous cell carcinoma (LUSC).

**Methods:**

This study retrospectively evaluated the patients with advanced LUSC treated with the combination of radiotherapy with immunotherapy and chemotherapy (ICRT group, n = 52) or immunotherapy and chemotherapy (ICT group, n = 63) as the first-line treatment from April 2018 to April 2022. Using propensity score matching (PSM), 50 pairs were created, while the confounders and bias were controlled. The objective response rate (ORR), duration of overall response (DOR), progression-free survival (PFS), overall survival (OS), and adverse events were analyzed in the two groups. The PFS and OS were re-analyzed separately for patients treated with thoracic radiotherapy.

**Results:**

After PSM, the median PFS (12.23 *vs.* 7.43 months; *P <*0.001) and median OS (19.7 *vs.* 12.9 months; *P <*0.001) were significantly longer in the ICRT group than those in the ICT group. Both the PFS and OS rates were also significantly higher in the ICRT group than those in the ICT group, except for the OS rates in the 6th and 12th months. The mDOR of the ICRT group patients (17.10 *vs.* 8.27 months; *P <*0.001) was significantly higher than that of the ICT group patients. The median PFS, median OS, and local control rate were significantly longer in the thoracic radiotherapy group than in the control group. Radiation pneumonia was the most common adverse effect after radiotherapy; however, no treatment-related deaths occurred. The Cox regression analysis showed that ECOG scores 0-1, presence of necrosis in the tumor, radiotherapy, and optimal efficacy better than the stable disease (SD) were independent factors, affecting the PFS, while the patients with recurrent post-operative, pre-treatment NLR, radiotherapy, and optimal efficacy better than SD were the independent factors, affecting the OS.

**Conclusions:**

The combination of radiotherapy with systematic immunotherapy and chemotherapy for the advanced LUSC was effective with tolerable adverse effects.

## 1 Introduction

According to GLOBOCAN 2022 (1), lung carcinoma is the most common malignant tumor and a major reason for cancer-related deaths worldwide. Almost 75% of lung cancers are non-small cell lung cancer (NSCLC), while 70% of them were already in the advanced stages at the time of diagnosis. Lung squamous cell carcinoma (LUSC) is an important pathological type of NSCLC, accounting for approximately 30% of all lung cancer patients. As compared to those with lung adenocarcinoma (LUAD), patients with LUSC have a higher frequency of somatic mutations and are more susceptible to multiple genetic mutations, leading to a worse prognosis. Immune checkpoint inhibitors (ICIs) have evolved from second-line to first-line treatment and have also been used in combination therapies for advanced LUSC in recent years. ICIs play important roles in improving the patient’s outcome, thereby bringing hope to the patients with advanced LUSC.

The KEYNOTE-024 (2) used ICIs for the advanced NSCLC and confirmed the superiority of first-line programmed cell death protein-1(PD-1) inhibitor monotherapy over conventional chemotherapy in ≥ 50% PD-L1-positive NSCLC patients. The KEYNOTE-042 (3) and KEYNOTE-407 (4) studies emphasized the importance of ICIs monotherapy for PD-L1-positive NSCLC patients and the combination of ICIs and chemotherapy for advanced LUSC patients. Rational 307, Orient 12, Camel-sq, CheckMate-227, CheckMate-9LA, Impower110, and GEMSTONE-302 studies (5–11) have suggested various options for advanced LUSC patients. As compared to LUAD patients, advanced LUSC patients have a higher ORR and shorter PFS and OS rates in first-line immunotherapy. In an extension study for KEYNOTE-407 in China (12), the ORR of pembrolizumab in combination with chemotherapy was 80%; however, the median PFS (mPFS) was only 8.3 months. Though the combination of sintilimab and chemotherapy reduced the risk of progression (38%) in the Orient 12 study, the mPFS was only 5.1 months. Therefore, studies, exploring the strategies for prolonging the mPFS of advanced LUSC patients undergoing first-line immunotherapy, are urgently needed.

Among previous explorations, the combination of radiotherapy and immunotherapy has created a synergistic effect (13). First, radiation causes tumor cell death, producing tumor-associated antigens and causing *in situ* vaccination effects (14). Second, radiation induces intracellular stress, causes the occurrence of immunogenic cell death, promotes the cross-presentation of dendritic cells (DCs) antigens, induces the local immune response, and actives the CD8+T lymphocyte, thereby transforming “cold” tumors into “hot” tumors (15). Then, improving the tumor immune microenvironment might partially overcome the resistance of poorly immunogenic tumors to anti-PD-1/PD-L1 inhibitors. Third, some studies suggested that a high tumor burden could decrease the effects of immunotherapy. Radiation eliminates cancers directly or indirectly, relieving the immunosuppressive state for patients and even enhancing the PD-L1 expression, which further improves the efficacy of ICIs. Finally, radiation reduces the accumulation of local tumor-infiltrating myeloid-derived suppressor cells. Moreover, CD8+ T cells were stimulated by the combination with PD-1 inhibitors and radiotherapy, which might increase the Tumor Necrosis Factor-α (TNF-α) release at the same time, thereby further eliminating the tumor-infiltrating myeloid-derived suppressor cells and establishing a long-term anti-tumor effect (16).

In the past, studies, investigating the combination of radiotherapy and immunotherapy, have mainly focused on the inoperable locally advanced NSCLC. The PACIFIC, Lung14-179, GEMSTONE-301, and DETERRED studies (17–20) showed that the combination of synchronous or sequential radiotherapy with the anti-PD-1/PD-L1 inhibitors could improve the efficacy of locally advanced NSCLC. However, the efficacies of ICIs with or without radiotherapy in the advanced NSCLC (especially LUSC) have rarely been reported. Moreover, the lesions in LUSC patients are mostly located up to the segmental bronchi, which can frequently cause clinical symptoms, such as cough, hemoptysis, and pulmonary atelectasis. More importantly, the symptoms caused by local lesions might partially persist after systemic treatment, thereby affecting the quality of life. In addition, after immunotherapy, the risk of local recurrence is higher as compared to the progression of the disease anywhere else in the advanced NSCLC (13, 21). Apparently, radiotherapy has been preferably used to alleviate the clinical symptoms and reduce the risk of local recurrence at the same time. Therefore, this retrospective study mainly aimed to investigate the combination treatment effects of radiotherapy, systematic immunotherapy, and chemotherapy, in order to preliminarily explore an optimal treatment model for the combination treatment of advanced LUSC patients.

## 2 Objects and methods

### 2.1 Objects

#### 2.1.1 Patients

The patients with advanced LUSC (recurrent or metastatic LUSC), who were treated with platinum-based chemotherapy and ICIs as first-line treatment in the Second Affiliated Hospital of Chongqing Medical University, Chongqing, China, from April 2018 to April 2022 were recruited in this study. Based on receiving radiotherapy after achieving disease control by the first-line treatment, the patients were divided into the immunotherapy + chemotherapy + radiotherapy (ICRT) group and the immunotherapy + chemotherapy (ICT) group. Besides ICRT and ICT groups, a subgroup analysis was also performed. The subgroups included thoracic radiotherapy group and control group. The thoracic radiotherapy group included the patients receiving thoracic radiotherapy (a subgroup of ICRT group), while the control group included the patients treated without radiotherapy (a subgroup of ICT group).

#### 2.1.2 Inclusion criteria

(1) The patients, who were diagnosed with LUSC based on the diagnostic criteria of the Chinese Society of Clinical Oncology for Primary Lung Cancer (2018 Edition); (2) the patients, who received platinum-based chemotherapy and PD-1 immunotherapy as the first-line treatment; (3) the patients with Eastern Cooperative Oncology Group (ECOG) scores of ≤2.

#### 2.1.3 Exclusion criteria

(1) The patients with ECOG score >2; (2) the patients with the uncontrolled disease after first-line treatment; (3) the patients having other primary tumors; (4) the patients having other serious primary diseases.

### 2.2 Methods

#### 2.2.1 Treatments

The patients in both groups received the combination of platinum-based chemotherapy and ICIs as the first-line treatment. Radiotherapy was performed for the ICRT group patients after controlling the disease. Then, the ICI maintenance treatment (pembrolizumab, sintilimab, camrelizumab, or tislelizumab 200 mg every 21 days; toripalimab 240 mg every 21 days) was performed for a maximum of 35 cycles. In contrast, after 4–6 cycles of the combined chemotherapy and ICIs, the control group was also given the same ICI maintenance treatment. In order to ensure the patient’s tolerance to the treatment, the ICI treatments were delayed or interrupted briefly upon the occurrence of ≥grade 2 clinical toxicity according to Common Terminology Criteria for Adverse Events (CTCAE) version 5.0. After disease progression, second-line chemotherapy, ICIs, multi-targeted drugs, local intervention (radiotherapy, transcatheter arterial chemoembolization), or supportive care were performed.

#### 2.2.2 Observation of indexes

The peripheral venous blood samples were collected from the patients before three days of first-line treatment, and the neutrophils to lymphocytes ratio (NLR), C-reactive protein (CRP) levels, lactate dehydrogenase (LDH) levels were identified. After treatments, the patients were followed up, and enhanced computed tomography (CT) or brain magnetic resonance imaging (MRI) were performed once every 1-2 months at first for six months and then once every two months afterward for 4 years. All the patients were evaluated based on RECIST1.1 criteria. PFS was defined as the time from the first-line treatment to the first occurrence of tumor progression, death, or the end of follow-up. OS was defined as the time from the first-line treatment till death or the end of follow-up. The optimal duration of response (DOR) was defined as the time from the complete response (CR) or partial response (PR) to initial progressive disease (PD), death, or the end of follow-up. Clinically toxicities were assessed according to CTCAE, version 5.0.

#### 2.2.3 Statistical analyses

The potential confounders and selection bias were controlled using propensity score matching (PSM). The data of patients, including age, ECOG score, postoperative recurrence, extrapulmonary or bone metastases, NLR, presence of cavities or necrosis within the tumors, and the sum of the target lesion were collected and entered into the PSM model, which was used to provide one-to-one matches using the nearest-neighbor method. The continuous and categorical variables between the two groups before and after PSM were analyzed using Student’s *t*-test and chi-squared test, respectively.

All the statistical analyses were performed using SPSS version 26 and R Programming Language version 4.2.1. The survival analysis was performed using Kaplan–Meier survival curve analysis and the Log-rank test. Then, the univariate and multivariate cox regression analyses were performed at the same time using Cox Regression Model. A *P*-value of <0.05 was considered statistically significant. The variables, having a *P*-value of <0.1 in the univariable analysis were screened into the Cox Regression analysis.

## 3 Results

### 3.1 Baseline characteristics

A total of 115 patients were included in this study, among which, 52 patients received the combination of radiotherapy, a PD-1 inhibitor, and chemotherapy (ICRT group), while the rest (n = 63) received the combination of PD-1 inhibitor and chemotherapy only (ICT). The characteristics of all the patients were listed in **Table 1**. The patients in the ICRT group showed a higher probability of postoperative recurrence (44.4% *vs.* 18.9%; *P* = 0.057) as compared to those in the ICT group, before matching. There were no significant differences in the age, sex, ECOG score, smoking, extrapulmonary/intrapulmonary or bone metastases, PD-L1 status, NLR, LDH, CRP, presence of cavitation or necrosis, the sum of the target lesion, and optimal efficacy for the patients achieving CR/PR between two groups (*P >*0.05). Among the total 115 patients, PD-L1 testing was performed for 64 patients, among which, 21 patients had PD-L1 ≥1% (1%-90%), while the rest of the patients were PD-L1-negative, showing no sensitive gene mutations. After PSM, the clinical characteristics of the patients were more balanced. Moreover, according to the irradiated sites, 34 patients were selected as the thoracic radiotherapy group, and PSM was performed. Then, 34 patients, who did not undergo thoracic radiotherapy, were selected as the matching control group. The clinical characteristics for these 68 patients were balanced, showing no significant difference between the two groups before or after matching (*P >*0.05) (**Table 2**).

**Table 1:** Baseline characteristics of all the patients.

Characteristic	Before Matching			After Matching		
	ICRT Group	ICT Group	*P* value	ICRT Group	ICT Group	*P* value
	(n=52)	(n=63)		(n=50)	(n=50)	
Age (x ± s, years)	64.31 ± 8.39	66.54 ± 8.75	0.169	64.66 ± 7.76	66.02 ± 9.23	0.427
Gender (female/male)	6/46	8/55	0.850	5/45	7/43	0.538
ECOG (0-1/2)	24/28	32/31	0.620	23/27	22/28	0.841
Smoke (<400/≥400)	18/34	25/38	0.576	17/33	21/29	0.410
Extrapulmonary metastases (No/Yes)	27/25	33/30	0.961	27/23	26/24	0.841
Intrapulmonary metastasis (No/Yes)	42/10	44/19	0.179	40/10	34/16	0.171
Bone metastasis (No/Yes)	39/13	44/19	0.539	38/12	34/16	0.373
Postoperative recurrence (No/Yes)	36/16	53/10	0.057	34/16	40/10	0.171
Cavitation or necrosis (No/Yes)	33/19	35/28	0.391	33/17	30/20	0.534
PD-L1 Status						
Negative	20	23	0.829	20	18	0.680
Positive	10	11	0.807	10	10	1.000
Unknown	22	29	0.689	20	22	0.685
CRP (x ± s, mg/L)	36.36 ± 43.95	43.30 ± 49.65	0.434	35.77 ± 44.42	40.42 ± 42.33	0.594
LDH (x ± s, U/L)	200.79 ± 55.29	192.21 ± 42.09	0.347	201.46 ± 56.00	189.56 ± 41.12	0.229
NLR (x ± s)	6.02 ± 5.29	6.60 ± 6.09	0.589	5.61 ± 4.94	6.01 ± 4.69	0.679
Sum of target lesions(x ± s, mm)	58.09 ± 36.30	64.02 ± 25.05	0.321	57.31 ± 36.54	60.20 ± 24.22	0.643
Optimal response to CR/PR (No/Yes)	20/32	33/30	0.136	20/30	26/24	0.229

The ICRT group showed a higher probability of postoperative recurrence (44.4% vs. 18.9%; P = 0.057) as compared to the ICT group, before matching. The differences in the age, sex, ECOG score, smoking status, extrapulmonary/intrapulmonary or bone metastases, PD-L1 status, NLR, LDH, CRP, presence of cavitation or necrosis, the sum of the target lesion, and the optimal efficacy of the patients who achieved CR/PR between two groups were not significant (P >0.05).

**Table 2:** Baseline characteristics of patients.

Characteristic	Before Matching				After Matching			
	Thoracic Radiotherapy Group	ICT Group	*P* value		Thoracic Radiotherapy Group	Control Group		*P* value
	(n=34)	(n=63)			(n=34)	(n=34)		
Age (x ± s, years)	64.65 ± 7.73	66.54 ± 8.75	0.293		64.65 ± 7.73	67.65 ± 9.41		0.156
Gender (female/male)	3/31	8/55	0.811		3/31	5/29	0.707	
ECOG (0-1/2)	17/17	32/31	0.941		17/17	12/22	0.220	
Smoke (<400/≥400)	12/22	25/38	0.671		12/22	14/20	0.618	
Extrapulmonary metastases (No/Yes)	22/12	33/30	0.242		22/12	24/10	0.604	
Intrapulmonary metastasis (No/Yes)	24/10	44/19	0.939		24/10	21/13	0.442	
Bone metastasis (No/Yes)	28/6	44/19	0.179		28/6	27/7	0.758	
Postoperative recurrence (No/Yes)	25/9	53/10	0.210		25/9	27/7	0.567	
Cavitation or necrosis (No/Yes)	24/14	35/28	0.757		24/14	24/14	1.000	
PD-L1 Status								
Negative	12	23	0.905		12	13	0.801	
Positive	8	11	0.472		8	8	1.000	
Unknown	14	29	0.646		14	13	0.804	
CRP (x ± s, mg/L)	37.49 ± 46.21	43.30 ± 49.65		0.575	37.49 ± 46.21	44.38 ± 48.14	0.549	
LDH (x ± s, U/L)	197.03 ± 52.19	192.21 ± 42.09		0.622	197.03 ± 52.19	194.74 ± 43.69	0.845	
NLR (x ± s)	5.54 ± 4.93	6.60 ± 6.09	0.380		5.54 ± 4.93	6.41 ± 5.38	0.487	
Sum of target lesions(x ± s,mm)	62.04 ± 37.64	64.02 ± 25.05	0.784		62.04 ± 37.64	57.22 ± 22.06	0.522	
Optimal response to CR/PR (No/Yes)	12/22	33/30	0.107		12/22	18/16	0.143	

There were no significant differences in the age, sex, ECOG score, smoking status, extrapulmonary/intrapulmonary or bone metastases, PD-L1 status, NLR, LDH, CRP, presence of cavitation or necrosis, the sum of the target lesions, and the optimal efficacy of the patients who achieved CR/PR between the two groups (P >0.05).

The patients were treated with PD-1 inhibitors, including camrelizumab (15 cases), sintilimab (55 cases), pembrolizumab (5 cases), tislelizumab (21 cases), and toripalimab (19 cases) for a median time of 4.9 months (7 times). The chemotherapeutic drugs included paclitaxel analogs, docetaxel, gemcitabine, vinorelbine, and platinum analogs. The median cycles of chemotherapy were 4 cycles for the first-line treatment. The irradiated sites in the radiotherapy group included thoracic (34), liver (3), bone (7), brain (2), lymph nodes (7), and adrenal gland (1) sites. The irradiated targets were gross tumor volume (GTV) for the thoracic or metastatic radiotherapy and elective nodal irradiation (ENI) for positive lymph nodes. Radiotherapy was given at a dose of 2-5 Gy per fraction with a total dose of 45-60 Gy for GTV/ENI and 58.5-84 Gy for BED (**Table 3**).

**Table 3:** Dose and local control rate of the ICRT group.

	Physical Dose (Gy)	Biological Effect Dose (Gy)	Local Control Rate(%)
Conventional radiation for Thoracic (19 cases)	60Gy/30F/2Gy	72Gy	89.5%
Hypo-fractionated radiation for Thoracic (11 cases)	60Gy/15F/4Gy	84Gy	81.8%
Hypo-fractionated radiation for Thoracic (4 cases)	50Gy/10F/5Gy	75Gy	75%
Elective Nodal radiation (7 cases)	60Gy/30F/2Gy	72Gy	57.1%
Hypo-fractionated radiation for Bone metastases (7 cases)	45Gy/15F/3Gy	58.5Gy	71.4%
Hypo-fractionated radiation for Liver metastases (3 cases)	45Gy/15F/3Gy	58.5Gy	33.3%
Hypo-fractionated radiation for Brain metastases (2 cases)	51Gy/17F/3Gy	66.3Gy	100%
Conventional radiation for Adrenal gland metastases (1 case)	50Gy/25F/2Gy	60Gy	0%

### 3.2 Effectiveness and safety

Among the total patients, 83.5% of patients achieved PFS, while 60% of the patients achieved OS terminal. The median follow-up time was 14.5 months. Before matching, the median PFS (12.23 *vs.* 6.7 months; Hazard Ratio, HR 0.40, *P <*0.001) and median OS (19.7 *vs.* 11.33 months; HR 0.45, *P <*0.001) of the patients in the ICRT group were significantly longer as compared to those in the ICT group. The PFS rates of the patients in the ICRT group at the 3^rd^, 6^th^, and 12^th^ months were all significantly higher than those in the ICT group (*P <*0.001). As compared to the ICT group, the OS rates of the patients in the ICRT group at the 6^th^, 12^th^, and 18^th^ months were higher (*P <*0.05 for the 12^th^ and 18^th^ months, showing a significant difference). The efficacy evaluation showed that 5 and 27 patients achieved CR and PR, respectively, in the ICRT group. While 1 and 29 patients achieved CR and PR, respectively, in the ICT group. There were no significant differences in CR rate and ORR between the two groups (*P >*0.05) (**Table 4**).

**Table 4:** Survival analysis and therapeutic effects of the two groups.

Index	Before Matching			After Matching		
	ICRT Group	ICT Group	*P* value	ICRT Group	ICT Group	*P* value
	(n=52)	(n=63)		(n=50)	(n=50)	
mPFS (month) (95% CI)	12.23 (8.50-15.96)	6.7 (5.31-8.08)	<0.001	12.23 (8.37-16.09)	7.43 (6.44-8.42)	<0.001
PFS rate (%)						
3 month	98.1	81.0	0.004	98.0	80.0	0.004
6 month	84.6	57.1	0.001	84.0	66.0	0.038
12 month	51.8	13.3	<0.001	52.2	14.7	<0.001
mOS (month) (95% CI)	19.70 (13.69-25.71)	11.33 (8.11-14.55)	<0.001	19.70 (14.62-24.78)	12.90 (9.29-16.52)	<0.01
OS rate(%)						
6 month	94.2	84.1	0.089	94.0	88.0	0.487
12 month	69.6	48.8	0.017	68.3	54.7	0.113
18 month	56.1	20.1	<0.001	54.4	23.1	0.001
m Dor (month) (95% CI)	15.53 (10.26-20.79)	7.80 (6.90-8.69)	<0.001	17.10 (11.99-22.20)	8.27 (6.81-9.72)	0.001
Efficacy (cases)						
CR	5	1	0.090	5	0	0.056
PR	27	29	0.529	25	24	0.841
ORR(%)	61.5	47.6	0.136	60.0	48.0	0.229

After PSM, the median PFS and median OS were significantly longer in the ICRT group as compared to those in the ICT group (P <0.001). Both the PFS and OS rates in the ICRT group were significantly higher than those in the ICT group, except for the OS rains in the 6^th^ and 12^th^ months. The mDOR of the ICRT group was significantly higher than that of the ICT group (P <0.001).

**Figure 1:**
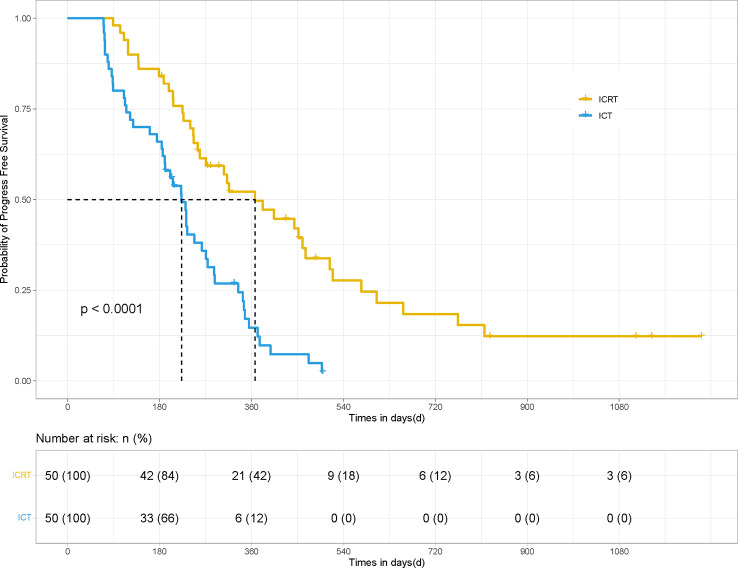
PFS of the patients in the ICRT and ICT groups for 100 patients. After PSM, the PFS results between the two groups were statistically significant (12.23 months *vs.* 7.43 months, HR 0.41, 95% CI [0.213, 0.534], *P <*0.001).

**Figure 2:**
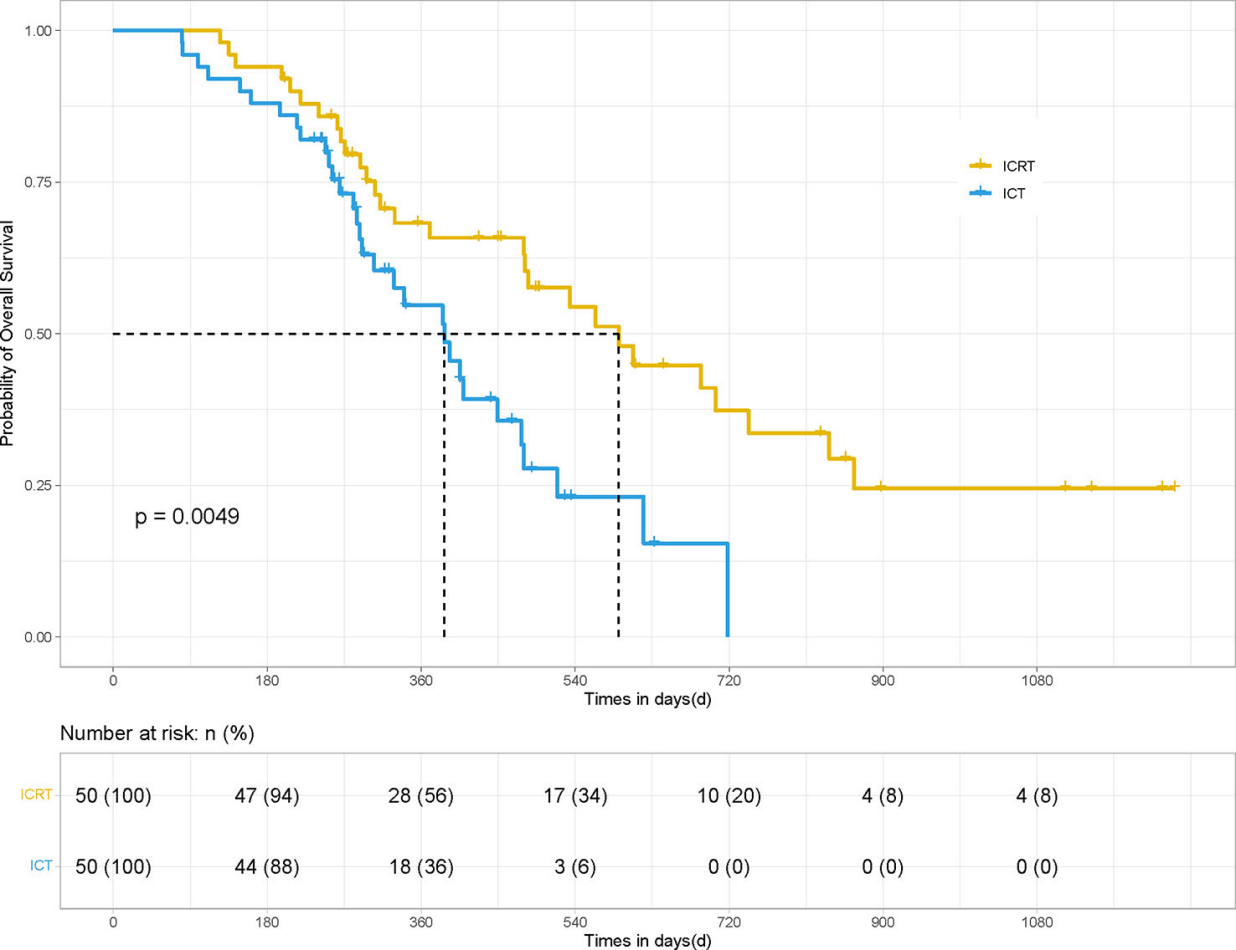
OS of the patients in the ICRT and ICT groups for 100 patients. After PSM, the OS results between the two groups were statistically different (19.7 *vs.* 12.9 months; HR 0.49, 95% CI [0.250, 0.729], *P <*0.01).

After PSM, the mPFS and OS of patients in the ICRT group were significantly better than those in the ICT group (for mPFS: 12.23 *vs.* 7.43 months; HR 0.41, 95% CI, Confidence Interval, [0.213, 0.534], *P <*0.001) (**Figure 1**) and for OS: 19.7 *vs.* 12.9 months; HR 0.49, 95% CI [0.250, 0.729], *P <*0.01) (**Figure 2**). Meanwhile, the PFS rates of the patients in the ICRT group at the 3^rd^, 6^th^, and 12^th^ months were higher than those in the ICT group (98%, 84%, and 52.2% *vs.* 80%, 66%, and 14.7%). The OS rates of the patients in the ICRT group at the 6^th^, 12^th^, and 18^th^ months were higher than those in the ICT group (94%, 68.3%, and 54.4% *vs.* 88%, 54.7%, and 48%). Both the PFS and OS rates of the patients in the ICRT group were significantly higher than those in the ICT group, except for the OS rates at the 6^th^ and 12^th^ months (*P >*0.05). There were no significant differences in the CR rate and ORR between the two groups (*P >*0.05), while the mDOR was statistically longer in the ICRT group as compared to that in the ICT group (17.10 *vs.* 8.27 months; *P* = 0.001) (**Table 4**).

**Figure 3:**
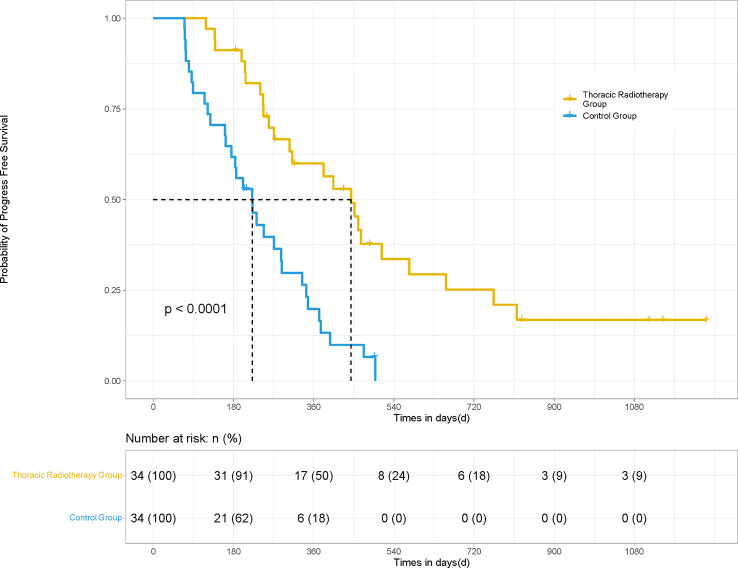
PFS of the patients in the thoracic radiotherapy and control group for 68 patients. For those patients treated with thoracic radiotherapy, PSM was performed, and 34 patients without radiotherapy were matched for comparison. The mPFS (14.8 *vs.* 7.4 months; HR 0.36, 95% CI [0.163, 0.507], *P <*0.001) of the patients in the thoracic radiotherapy group was significantly higher than those in the control group.

**Figure 4:**
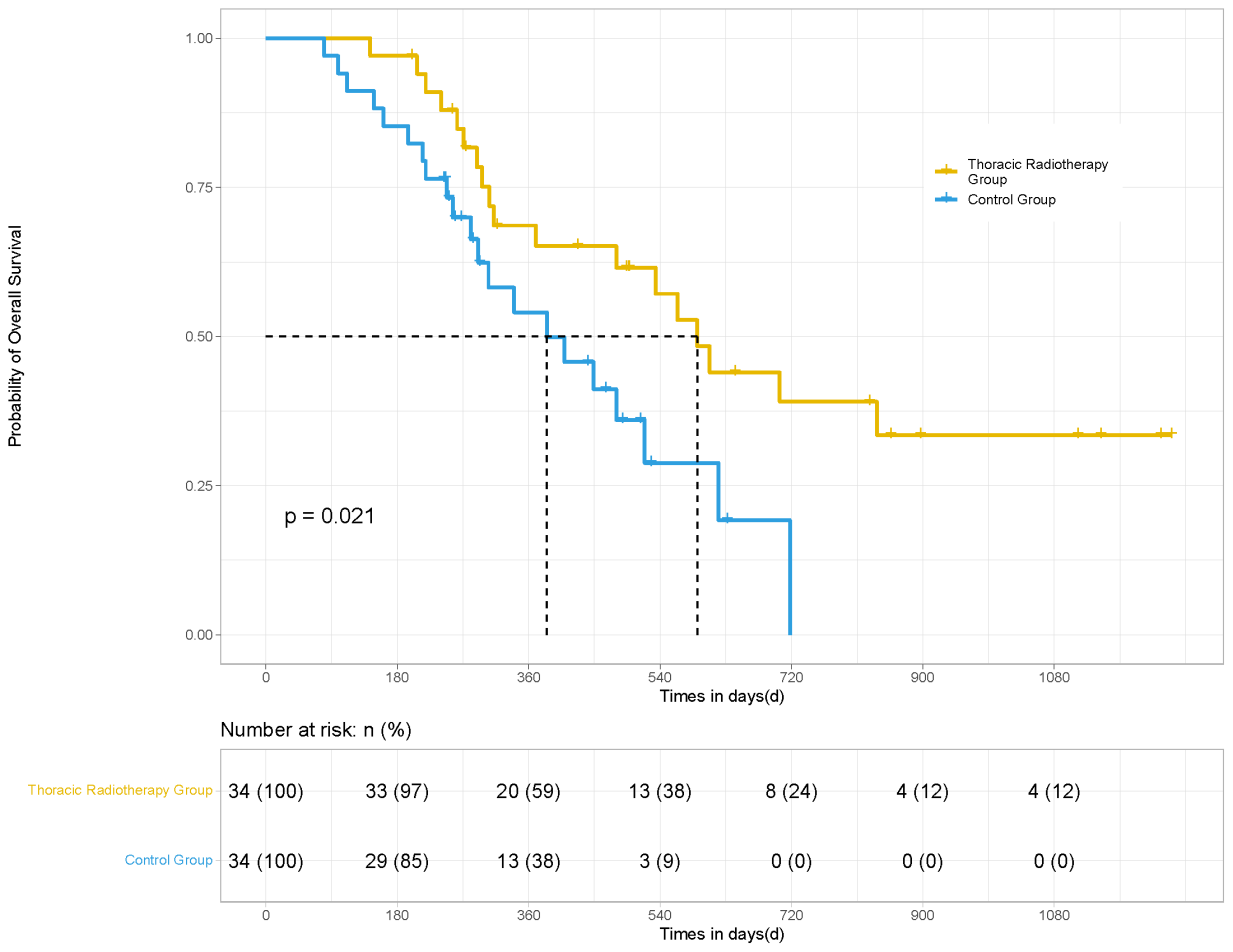
OS of the patients in the thoracic radiotherapy and control group for 68 patients. After PSM, the mOS (19.7 *vs.* 12.8 months; HR 0.50, 95% CI [0.235, 0.865], *p* = 0.021) of the patients in the thoracic radiotherapy group was significantly higher than those in the control group.

In terms of irradiated sites, 34 patients underwent thoracic radiotherapy for their pulmonary lesions. PSM was performed for the patients in the thoracic radiotherapy group in a 1:1 ratio (34 matching control patients without radiotherapy). The mPFS (14.8 *vs.* 7.4 months; HR 0.36, 95% CI [0.163, 0.507], *P <*0.001) and mOS (19.7 *vs.* 12.8 months; HR 0.50, 95% CI [0.235, 0.865], *P* = 0.021) of the patients in the thoracic radiotherapy group were significantly higher than those in the control group (**Figures 3**, **4**).

The disease progression model showed that a total of 37 patients exhibited progressed diseases in the ICRT group; 11 of them recurred in the irradiated sites, while the rest progressed in other sites. The local recurrence rate in the ICRT group patients was 22%. A total of 45 patients exhibited PFS events in the ICT group; 29 of them recurred in the primary pulmonary lesions with a recurrence rate of 58%. Meanwhile, 34 patients suffered from cough, superior vena cava syndrome, pain, etc. before radiotherapy. After radiotherapy, most of these symptoms (88.2%) were well-alleviated. In the thoracic radiotherapy group, 24 patients exhibited PFS events, such as bone metastasis (4), lymph node failure (4), brain metastasis (3), malignant pleural effusion (4), bronchial progression (1), malignant pericardial effusion (1), adrenal gland metastases (1), and death (3). Only 3 of these events progressed in the primary pulmonary site. The recurrence rate in the thoracic radiotherapy group was 8.8%. In contrast, 31 patients showed PFS events in the control group, among which, 20 events recurred at the primary pulmonary lesions. The recurrence rate in the control group was 58.8%, which was significantly different from that in the thoracic radiotherapy group (*P <*0.05).

**Figure 5:**
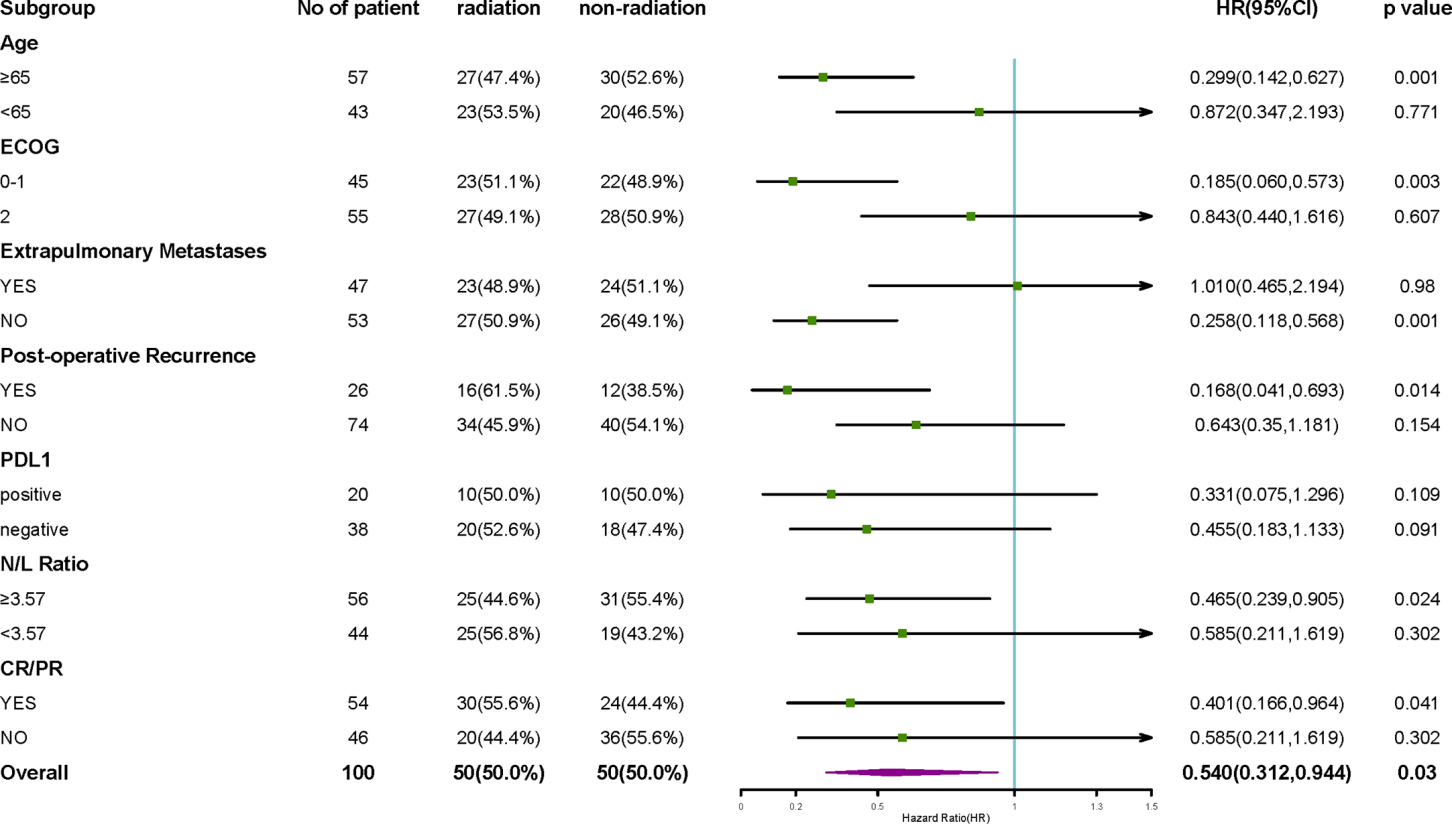
Subgroup analysis of OS. The subgroup analysis of OS suggested that the efficacy of radiotherapy combined with ICIs was the most favorable among patients with ages ≥65 years, patients with ECOG score 0-1, patients without extrapulmonary metastasis, patients who had a postoperative recurrence, patients with NLR ≥3.57, and the patients, which achieved CR/PR from the first-line treatment. The effects of PD-L1 expression on radiotherapy benefits are not clear.

In the subgroup analysis, the combined efficacy of radiotherapy and ICIs was the most favorable for the following patients: the patients with ages ≥ 65 years (*P*  = 0.001); the patients with ECOG scores 0-1 (*P*  = 0.003); the patients without extrapulmonary metastasis (*P*  = 0.001); the patients, who had postoperative recurrence (*P*  = 0.014); the patients with NLR ≥3.57 (*P*  = 0.024); and the patients showing CR/PR to the first-line treatment (*P*  = 0.041) (**Figure 5**). Radiotherapy showed a trend of having greater clinical benefits in the PD-L1-positive subgroup than in the PD-L1-negative subgroup, even though half of the patients’ PD-L1 statuses were unknown.

In terms of safety, after chemotherapy combined with PD-1 immunotherapy, the most common adverse effects included leukocytopenia and thrombocytopenia; no treatment-related deaths occurred in the two groups. However, there were other adverse effects, such as peripheral neuritis (3), transaminase elevation (20), thyroid dysfunction (12), immunotherapy-related myocarditis (4), checkpoint inhibitors pneumonitis (CIP) (12), and rash (7). There were no significant differences in all the treatment-related adverse effects between the two groups (**Table 5**). Radiation pneumonitis (17), esophagitis (5), and hepatitis (1) were unique to the ICRT group, which occurred within six months after radiotherapy and were recovered after hormonal, antibiotic, nutritional support, and symptomatic treatments. Grade 3 radiation-related adverse effects, including radiation pneumonitis (2), and esophagitis (1), were unique to the ICRT group.

**Table 5:** Safety assessment of two groups.

Toxic effects	total (n=100)	ICRT Group (n=50)	ICT Group (n=50)	*p* value
Leukopenia	55	26	29	0.546
Grade 1-2/case	20	9	11	
Grade 3-4/case	35	17	18	0.799
Thrombocytopenia	47	23	24	0.841
Grade 1-2/case	24	13	11	
Grade 3-4/case	23	10	13	0.464
Transaminase elevation	20	9	11	0.617
Grade 1-2/case	18	8	10	
Grade 3-4/case	2	1	1	1.000
Thyroid dysfunction	12	7	5	0.538
Grade 1-2/case	10	6	4	
Grade 3-4/case	2	1	1	1.000
Immunotherapy-related myocarditis	4	1	3	0.617
Grade 1-2/case	2	1	1	
Grade 3-4/case	2	0	2	1.000
Checkpoint inhibitors pneumonia	12	6	6	1.000
Grade 1-2/case	4	3	1	
Grade 3-4/case	8	3	5	0.545
Peripheral neuritis	3	2	1	1.000
Drug-induced rash	7	5	2	0.433
Radiation pneumonia	17	17	/	<0.001
Grade 1-2/case	15	15	/	<0.001
Grade 3-4/case	2	2	/	<0.001
Radiation esophagitis	5	5	/	<0.001
Radiation hepatitis	1	1	/	<0.001

The most common adverse effects after the combination of chemotherapy and PD-1 immunotherapy included leukocytopenia and thrombocytopenia. No significant difference was found between the two groups for all the treatment-related adverse effects, including peripheral neuritis, transaminase elevation, thyroid dysfunction, immunotherapy-related myocarditis, checkpoint inhibitors pneumonia, and rash. The unique adverse effects of the patients in the ICRT group were pneumonia, esophagitis, hepatitis, etc., which could be recovered after hormonal, antibiotic, nutritional support, and symptomatic treatments. Grade 3 radiation-related adverse effects, including pneumonia and esophagitis, were unique to the ICRT group.

### 3.3 Factors affecting PFS and OS

Univariate analysis was performed on age, sex, ECOG score, smoking, extrapulmonary/intrapulmonary or bone metastases, PD-L1 status, NLR, LDH, CRP, presence of cavitation or necrosis, the sum of the target lesion, and CR/PR of the patients. The results illustrated that the ECOG score (*P* = 0.000), patients with postoperative recurrence (*P* = 0.027), extrapulmonary metastasis (*P* = 0.048), NLR (*P* = 0.013), cavitation or necrosis within the lesion (*P* = 0.045), optimal efficacy better than the stable disease (SD) (*P* = 0.000), and radiotherapy (*P* = 0.000) were the factors, significantly affecting PFS (*P <*0.05). Cox regression analysis was conducted by the input method. All these factors were screened based on *P <*0.1. The Cox regression analysis revealed that the ECOG score of 0-1 (regression coefficient -0.647, Wald value 6.084, *P* = 0.014, HR 0.52, 95% CI [0.313, 0.876]), presence of necrosis in the lesion (regression coefficient 0.516, Wald value 4.707, *P* = 0.030, HR 1.67, 95% CI [1.051, 2.671]), a combination of radiotherapy (regression coefficient -0.679, Wald value 7.026, *P* = 0.008, HR 0.50, 95% CI [0.307, 0.838]), and optimal efficacy better than SD (regression coefficient -0.827, Wald value 11.645, *P* = 0.001, HR 0.437, 95% CI [0.272, 0.703]) were the independent factors, affecting PFS in univariable analysis using the forward linear regression method.

The univariate analysis illustrated that the ECOG score (*P* = 0.000), postoperative recurrence (*P* = 0.013), NLR (*P* = 0.009), optimal efficacy better than SD (*P* = 0.000), and radiotherapy (*P* = 0.006) were the factors, affecting OS (*P* < 0.05). All these factors were screened based on *P <*0.1. The Cox regression analysis indicated that the postoperative recurrence (regression coefficient -0.822, Wald value 6.623, *P* = 0.010, HR 0.44, 95% CI [0.235, 0.882]), NLR (regression coefficient 0.060, Wald value 7.013, *P* = 0.008, HR 1.062, 95% CI [1.016, 1.111]), optimal efficacy better than SD (regression coefficient -1.024, Wald value 12.14, *P* = 0.000, HR 0.359, 95% CI [0.202, 0.639]), and radiotherapy (regression coefficient -0.612, Wald value 4.686, *P* = 0.030, HR 0.54, 95% CI [0.312, 0.944]) were the independent factors, affecting the OS of patients.

## 4 Discussion

The applications of ICIs in treating advanced NSCLC have been developed rapidly in recent years, showing inspiring therapeutic efficacy. However, the response rate of ICIs monotherapy in advanced NSCLC is only 30%. Its combination with chemotherapy can improve ORR to approximately 45%-68%. However, this combination can improve PFS by less than 4 months, especially for the advanced LUSC; as compared to chemotherapy alone, the combination of pembrolizumab and chemotherapy as the first-line treatment could prolong mPFS by 2.9 months in the KEYNOTE 407 study. The ORIENT-12 study showed that the combination of sintilimab with gemcitabine and platinum prolonged the mPFS by only 0.6 months. In the subgroup analysis of the Check-Mate 012 study (22), the combination of nivolumab and chemotherapy resulted in 33% ORR and 11.9 months mOS in the LUSC subgroup; this efficacy was inferior to that of the patients with LUAD. On the other hand, as compared to LUAD, LUSC is more likely to have local recurrence (21). Due to the specificity of its anatomical location, LUSC is mostly a central lung cancer. The symptoms, such as obstructive pneumonia, hemoptysis, cough, etc., might seriously affect the quality of life after recurrence. Currently, studies are needed explore possible ways for further prolonging the PFS of first-line treatment, delaying the local recurrence, and improving the quality of advanced LUSC patients’ lives. Among other options, the combination of radiotherapy and immunotherapy is a research hotspot.

The role of radiotherapy in NSCLC had been demonstrated in clinical practices, such as using stereotactic body radiation therapy (SBRT) for early-stage NSCLC, concurrent radiotherapy for locally advanced NSCLC, and palliative radiotherapy for advanced NSCLC. With the publication of PACIFIC and several subsequent studies, the PD-1/PDL-1 inhibitors in combination with concurrent/sequential radiotherapy have been recommended as the maintenance or concurrent treatment for the locally advanced NSCLC. While exploring the optimal treatment models for advanced NSCLC, the KEYNOTE-001 study (23) showed significantly longer PFS and OS in the patients treated with radiotherapy as compared to those, who were not treated with radiotherapy. PEMBRO-RT study (24), the first phase II randomized study, comparing the combination of SBRT and PD-1 inhibitor with PD-1 monotherapy, showed a better mPFS (6.6 *vs.* 1.9 months) and mOS (15.9 *vs.* 7.6 months) in the trial group; however, the difference was insignificant. The MDACC study (25) suggested that the combination of SBRT and PD-1 inhibitors could improve the ORR and mPFS (9.1 *vs.* 5.1 months) as compared to the control group. A pooled analysis (26) of two trials, involving 148 cases (n = 76 in the pembrolizumab monotherapy group and n = 72 cases in combined radiotherapy and pembrolizumab group), demonstrated a significant improvement in the primary endpoints, including abscopal response rate (41.7% *vs.* 19.7%; *P* = 0.0039) and abscopal control rate (65.3% *vs.* 43.4%; *P* = 0.0071). These trials have focused on radiotherapy as the second-line treatment. There is sufficient evidence for using the combination of ICIs and chemotherapy as first-line treatment; however, the studies on the role of radiotherapy are limited, thereby requiring further investigation.

This study focused on patients with advanced LUSC with recurrent or metastatic disease, who were treated with the combination of immunotherapy and chemotherapy ± radiotherapy as the first-line treatment. The patients were matched using PSM, and the appropriate population benefit from radiotherapy was analyzed using irradiated site analysis, subgroup analysis, and COX regression analysis. Due to the specificity of the tumor’s anatomical location and biological behavior, this study focused on LUSC and attempted to explore an optimal treatment model by combining radiotherapy with the first-line treatment. The results showed that after treatment with the combination of immunotherapy and chemotherapy for a few cycles, the addition of radiotherapy to the systematic treatment increased ORR while significantly improving the PFS, OS, and DOR with a 59% lower risk of disease progression and a 50% lower risk of OS events at the same time.

Li et al. (27) demonstrated at the World Congress of Lung Cancer (WCLC) 2022 that the combination of radiotherapy and sintilimab could prolong mPFS by 4 months and mOS by 14 months (16 *vs.* 30 months; *P <*0.05) among the patients with stage III-IV NSCLC. Ding et al. (28) also showed that performing radiotherapy along with the combined chemo-immunotherapy as the first-line treatment could improve the ORR (50.8% *vs.* 40% months) and mPFS (16.5 *vs.* 10.4 months; *P <*0.05) of advanced NSCLC patients as compared to those treated with systematic treatment only. The recruitment of patients for the Phase III NIRVANA-Lung Trial (29) (NCT03774732.) has been started, which will further evaluate the benefit of using radiotherapy for the advanced NSCLC. Thus, the current study preliminarily explored an optimal model for the combination of radiotherapy and first-line systemic treatment.

In order to explore the application time of radiotherapy combination, Bauml et al. (30) proposed a notable efficiency of radiotherapy as compared to historical controls using radical doses of radiation for all the lesions in combination with PD-1 inhibitors. However, patients with advanced NSCLC frequently suffered from a massive and extensive invasion tumor, multiple metastases, and even presence of malignant multi-plasmal effusions, which might limit the implementation of radiotherapy in the initial treatment. The Pembro-RT study (24) examined the effects of single-lesion SBRT in combination with pembrolizumab in advanced NSCLC patients. Although the PFS, OS, and ORR improved, the differences were insignificant. Therefore, the implementation of radiotherapy in initial treatment might be appropriate only for patients with oligometastasis. According to targeted molecular therapy (31), the optimal timing of administering combination of radiotherapy is when the continuous tumor regression less is than 5% as compared to the previous results of efficacy evaluation. A clinical study confirmed that the patient might be benefited from local treatment only if the patient’s disease was controlled by the first-line treatment (32). Therefore, in this study, radiotherapy was performed only if the evaluation of patients’ efficacy showed the achievement of stable disease or better. In this way, on the one hand, the abscopal control rate outside the irradiated field was enhanced. On the other hand, the volume of irradiated targets was reduced, the hypoxia-induced resistance to ionizing radiation was avoided, the local control rate was improved, and the occurrence of radiation-related adverse effects was reduced. In addition, to explore the timing schedule of maintenance after radiotherapy, a retrospective analysis showed that the administration of immunotherapy after 3 weeks of starting SBRT might result in a longer OS (19 *vs.* 15 months) as compared to that within 3 weeks (13). However, there is still no conclusive evidence for the timing schedule, thereby requiring large randomized controlled clinical trials.

In order to explore the sites and appropriate volume of irradiated targets, a subgroup analysis on 34 patients, receiving thoracic radiotherapy in comparison with matching 34 patients, receiving no thoracic radiotherapy, was performed. As a result, the risk of disease progression decreased by 64%, and the mPFS increased by 7.4 months, thereby showing potential superior benefits of thoracic radiotherapy as compared to radiation anywhere else. According to previous studies (33), approximately 60% of patients showed local recurrence after the combination of immunotherapy and chemotherapy first-line treatment for advanced NSCLC. LUSC patients were more likely to suffer from local recurrence than non- LUSC patients, implying that the administration of thoracic radiotherapy could improve local control. This was also confirmed indirectly by exploring the disease progression mode in the current study. The recurrence rate of the irradiated site’s progression was 22%, whereas that for the thoracic radiotherapy group was lower (8.8%). In contrast, 58.8% of patients in the control group showed local recurrence, which was significantly higher than those in the thoracic radiotherapy group. Thoracic radiotherapy enhanced the local control rate, reduced the risk of disease progression, and improved the prognosis for advanced LUSC. Meanwhile, the symptom remission rate was 88.2% after treatment in the radiotherapy group. However, in the thoracic radiotherapy group, almost 100% symptoms were alleviated, which also fully illustrated the necessity of thoracic radiotherapy.

The volume of irradiated targets was relatively limited in this study due to the patients’ metastatic diseases. In order to specify the volume of irradiated targets, the residual primary disease or metastases was selected after systematic treatment for GTV, and the positive lymph nodes were selected for ENI without a subclinical irradiated target. Some studies have been performed on the irradiation of lymphatic drainage as a subclinical target. Tumor-draining lymph nodes (TDLNs) are important places for the activation and aggregation of antitumor T cells, which might affect the acquired immune response (34). By altering the chemokine expression and CD8+ T cell trafficking, the irradiation of DLN might suppress the adaptive immune response. Therefore, using SBRT or ENI for TDLNs in combination with immunotherapy, the acquired immune response could be diminished in mouse models (35). Therefore, the TDLNs were not irradiated in this study.

For the dose and fraction size of radiation, hypo-fractionated radiotherapy (HFRT) has been widely used for NSCLC, especially for patients with a small volume of irradiated targets. A preclinical study showed (36) that HFRT might enhance the infiltration of cytotoxic CD8+T cells, leading to the upregulation of *Fas* or intercellular adhesion molecule (*ICAM*) gene expressions as well as the expression levels of tumor antigen peptides. Dewan MZ et al. (37) examined the effects of 8 Gy per fraction (3 fractions) radiotherapy in combination with anti-CTLA-4 (cytotoxic T lymphocyte-associated antigen-4) immunotherapy. The results showed abscopal effects of this dosing rather than using 20 Gy in a single dose. HFRT can induce the accumulation of endogenous cytoplasmic DNA, which activates the human stimulator of the interferon genes (hSTING) pathway (38). When a single dose of 12–18 Gy was administered, the expression of DNA exonuclease Trex1 increased significantly, leading to a decrease in the cytoplasmic double-stranded DNA, which was detrimental to the activation of the immune response (39). Therefore, a dose of 8–10 Gy per fraction for 1–2 fractions is currently considered an optimal dose for HFRT. Meanwhile, the PACIFIC study indicated that the patients benefited from the combination of conventional radiation regardless of the total dose. However, the dose and fraction size of radiation are still controversial due to the lack of randomized controlled trials. Due to the limited sample size in the current study, the subgroup analysis for studying different fraction sizes could not be performed. However, both conventional radiotherapy and HFRT showed better efficacy as compared to the control group. Meanwhile, the COX regression analysis suggested that the presence of cavitation/necrosis within tumors was identified as a significant risk factor for the disease progression, suggesting the requirement for a higher radiation dose. On the one hand, the excessive tumor growth accompanied by ischemic necrosis and cavity in the tumor center might always predict a worse malignancy and poorer prognosis. On the other hand, a massive tumor and ischemic necrosis might inevitably cause hypoxia in the central region, induce resistance to radiotherapy, and reduce local control.

In order to identify the appropriate population to combine radiation with the first-line treatment, the subgroup analysis of OS in the current study suggested that the combination of radiotherapy with the first-line treatment might benefit the patients with ages ≥65 years, ECOG scores of 0-1, NLR ≥3.57, post-operative recurrence, non-extra-pulmonary metastasis, or an optimal efficacy of CR/PR after systemic therapy. The COX regression analysis revealed that the better ECOG score was a protective factor for PFS. This might be explained by better adherence and tolerance to the treatment and a lower tumor burden, which are closely associated with the efficacy of PD-1 inhibitors (40). Therefore, local radiotherapy might further improve the long-term outcomes of patients with more favorable ECOG status.

In terms of NLR, the current study demonstrated that the patients with higher NLR (≥3.57) might benefit more from the combination of radiotherapy as compared to the patients with lower NLR (≤ 3.57). The COX regression analysis showed that a higher NLR might be an independent factor, resulting in poor prognostic. A pooled analysis (41) of clinical trials proposed that the NLR ≥3 could independently impair PFS and OS in the second-line immunotherapy of NSCLC patients. A clinical study compared the tumor immunophenotypes of patients with different NLRs and found that there were significantly higher numbers of CD8+ and PD-1+ immune cells and CD8+ and PD-1+ T cells infiltration into the tumor microenvironment along with a lower NLR (42). However, the underlying mechanism is still unknown. Therefore, patients with a higher NLR might require local radiotherapy to sensitize immunotherapy for improving their prognosis. Among other biomarkers, the effects of CRP and LDH on efficacy were also analyzed; however, no explicit evidence was found for either of them. Since the PD-L1 expression was not identified in more than half of the patients in this study, the exact effects require further investigation.

The patients with postoperative recurrence and no extrapulmonary metastasis achieved greater clinical efficacy from radiotherapy. The COX regression analysis indicated a 50% lower risk of OS in postoperative recurrence as compared to the patients with extrapulmonary metastasis. Due to the lower tumor burden and relatively confined volume of irradiated targets, the patients with postoperative recurrence could easily achieve disease control by the treatment without severe adverse effects. More importantly, radiotherapy and an optimal efficacy of CR/PR after systemic therapy were identified as the common independent factors, affecting the PFS and OS. This confirmed that the addition of radiotherapy might further improve the outcomes of advanced LUSC patients, who had achieved disease control by the combined immunotherapy and chemotherapy, especially for those patients, who gained PR or CR in the evaluation.

In terms of safety, the combination of radiotherapy with immunotherapy might affect the types and severity of treatment-related adverse effects, especially the occurrence of interstitial pneumonia, which has been of great clinical concern. Interstitial pneumonia had a higher incidence rate in the combination group of PEMBRO-RT as compared to that in the pembrolizumab group (8% *vs.* 26%), and 12 (17%) of them presented grade 3–5 immune-related adverse events (irAEs). A retrospective study (43) showed that there was no significant difference in the incidence of grade ≥2 irAEs, any grade of pneumonia, or grade ≥2 pneumonia between the radiotherapy and non-radiotherapy cohorts, which indicated that the combination therapy did not increase the risk of interstitial pneumonia during immunotherapy. According to LUN14-197, PACIFIC, and Gemstone 301 studies, during maintenance therapy, the incidence of interstitial pneumonia was slightly higher, and the risk of grade 3–4 interstitial pneumonia was only 3%–5.4% in the ICIs maintenance group as compared to that in the control group, showing an insignificant difference. Therefore, the combination of immunotherapy and radiotherapy did not significantly increase the incidence of interstitial pneumonia. In this study, the common adverse events included hematological toxicities after the chemotherapy combined with PD-1 immunotherapy, including leukocytopenia and thrombocytopenia, which could be recovered in a short time. There was no significant difference in the incidence of grade ≥3 adverse events or grade ≥3 interstitial pneumonia between the ICRT and ICT groups. Radiation pneumonia was the most frequent adverse effect in the radiotherapy group (50%); however, most of them were grade 1-2, and the incidence of grade 3 radiation pneumonia was only 5.8%, which recovered after treatment. The mediastinal lymph nodes were frequently treated by conventional radiotherapy instead of HFRT. There was one patient, suffering from grade 3 radiation esophagitis. Fortunately, after treatment with hormones, antibiotics, and esophageal absorptiometry, the patient’s symptoms were relieved at the end of the treatment. The irradiated volumes and sites might be notably related to this toxicity. Future studies are required to evaluate the efficacy and safety of different schedules.

Altogether, combination therapy is a promising strategy for advanced LUSC with tolerable side effects. However, this was a retrospective study with a limited sample size, and the confounders and biases were inevitable. The ORR of the ICRT group in this study was insignificantly better than that of the control group. This study did not clarify whether it was limited improvement in the short-term outcome of radiotherapy or the limited sample size of the study, which limited the potential benefits of the ORR. The fragmentation of irradiated sites also prevented the subgroup analysis of different irradiated targets and fraction sizes. In addition, among the PD-L1-positive patients, the subgroup analysis of the KEYNOTE 042 study showed that LUSC patients benefited more than non- LUSC patients. Based on this, the data of radiotherapy in combination with systemic treatment for LUSC has rarely been reported. Unfortunately, the discussion of this study was not sufficient due to the absence of PD-L1 status in half of the patients. The optimization strategies and benefits of adding radiotherapy to the first-line treatment require further investigation in randomized phase III clinical trials.

## 5 Conclusions

In conclusion, based on numerous preclinical and clinical studies, the combination of radiotherapy and immunotherapy might be an effective treatment modality for advanced NSCLC. Future studies should focus on the applications of different ICIs, dose and fraction sizes of radiation, appropriate volume and numbers of irradiated targets, and strategies of combination. This study suggested that the combination of radiotherapy with systematic immunotherapy and chemotherapy for the treatment of advanced LUSC might be safe and effective and might further improve the prognosis of patients.

## Data availability statement

The raw data supporting the conclusions of this article will be made available by the authors, without undue reservation.

## Ethics statement

The studies involving human participants were reviewed and approved by the Ethics Committee of the Second Affiliated Hospital of Chongqing Medical University. Written informed consent for participation was not required for this study in accordance with the national legislation and the institutional requirements.

## Author contributions

All authors participated in the design, interpretation of the studies, analysis of the data, and review of the manuscript. JQ, MZ and SY conducted the experiments. SY, XZ and HZ collected data. JQ, CZ and SY did data analysis, and JQ, ZY and YH wrote the manuscript. JQ 1† and SY1 †These authors contributed equally to this work and share first authorship. All authors contributed to the article and approved the submitted version.
